# Provings and Clinical Observations with High Potencies

**Published:** 1892-04

**Authors:** Malcolm Macfarlan

**Affiliations:** Philadelphia


### PROVINGS AND CLINICAL OBSERVATIONS WITH HIGH POTENCIES.

MALCOLM MACFARLAN, M. D., PHILADELPHIA.

(Continued from March number, page 100.

### SCUTELLARIA$^{40M}$.

Causes marked nervousness and restlessness. This for years has been a favorite remedy with me for nervous, hysterical women. It quickly calms them. They feel soothed, easy, and rested, and the most prominent symptom is better, sounder, and more refreshing sleep.

### SECALE-CORNUTUM$^{95M}$ (F.).

I have found this highly curative in *varicose ulcers* and enlarged veins of the lower extremities in old men and women where the ulcers have been of years' standing. Very useful in dry gangrene of the aged. It produces pains through the eyes, coming from over the forehead into his eyes, feels pains worse in his eyes than anywhere else; burning sensation in them. *Very thirsty* continually; soreness in the back of the neck; sensation as if he had been struck; had chills, never shook so in his life; chill from four A. M. until ten A. M.; chill worse in back. During chill severe muscular pains in extremities. Neither fever nor sweat followed the chill. Vomited during the chill a quantity of bile. Has had frontal headache for a week. Worse in the afternoon.

### SENEGA$^{5C}$.

Menses come on too soon. Gnawing pain in the left side at the waist. Nausea continually. Dry scraping sensation in pharynx. Hoarseness.

### SEPIA$^{45M}$.

Produced marked catarrhal conjunctivitis; quickly brought on the menses on a prover with amenorrhœa for five or six months; throbbing pains in top and back of the head. Left ovarian soreness and bearing-down sensation in the uterus. Have verified frequently the symptom of left ovarian soreness. Right ovary only slightly affected.

### Serpentaria$^{5C}$.

Quite dizzy and weak; would fall if she didn't take hold of something; symptoms of cold in chest with cough; caused an eruption like a variety of eczema in patches, dry, scurfy, indurated skin.

### Silicea$^{CM}$.

Frequently cured hang-nails, "run-arounds," ulceration about the nails, toe-nail ulcers, with thin, offensive discharges; causes sensation of *tightness* across the *abdomen;* throbbing at the pit of the stomach; difficulty of expelling the stool; rectum seems paralyzed.

Has often relieved pain in the left ovary. This symptom discovered accidentally while giving the remedy for other troubles.

### Spigelia.

Caused working, twitching, or spasmodic movements of eyelids. Nervous condition of body generally.

### Spongia-tost.$^{CM}$.

Watering of eyes; latterly gummy or mucous discharge, with obscured vision; dry cough; clearing of throat constantly. I have found it generally the most effective remedy in true as well as spasmodic croup.

### Strontia-carb.$^{5C}$.

Severe pain from the knees to the ends of the toes; abdomen much swollen; uncomfortable fullness of abdomen; slight show of menses ever since her last period.

Kind of a smothering as if she couldn't get her breath; distress about or around her heart, as if pressed upon; couldn't rest; moaned at night; diarrhœa of a very violent and persistent kind; on the vessel every few moments; griping at the lower part of her abdomen and in rectum; pain on urinating; perfectly easy after four in the morning.

Bowels that only moved twice a week now move three times daily. This continued as long as medicine was taken.

STANNUM$^{30M}$.

Provings on consumptives; occasionally through the day they were seized with a great weakness at epigastrium, and then felt hungry, but could not eat; sensation as if they would faint; empty feeling at epigastrium; symptoms entirely new to the provers.

STILLINGIA-SYL.$^{45M}$.

Fever at one o'clock P. M.; gets into a heavy sleep; perspiration toward morning; have not made a thorough proving of this remedy.

STRAMONIUM$^{CM}$.

Pain in loins; urine turbid, brown, and thick; very scanty; no force with it; proving was not pushed to extremes. This was the first symptom noticed.

SULPH-ACID$^{5C}$.

Soreness between the scapulæ; feels tired; tight, dry cough, slight hacking. Acted like magic in curing bronchitis in children with short, teasing cough. Frequently verified latter symptom.

SULPHUR$^{CM}$ and $^{45M}$.

The most effectual of all remedies I have ever used in the many forms of brain disease and in extreme cases with unconsciousness, stupor, mostly of children, continued low fever, cases which look like death when Bell., Zinc., Hellebore, and Hyos. were of no service, Sulphur helped promptly.

SULPHUR$^{2C}$.

*Heaviness of the legs and thighs;* so tired prover can scarcely walk; has to stop several times in walking up-stairs; caused emissions three times daily. This took place during the third week. Frequently cured seminal emissions. Cured many cases of otorrhœa after scarlet fever. Several cases of amenorrhœa in young girls with great debility. Parents fear the child is going into decline. Produces fearful hunger and craving for food, mostly for dinner. Can't go to bed or church without taking food with her. Caused turbid, yeasty urine in many of the cases. Great weakness in the legs. Can hardly go about. Wants to lie down. Great gnawing hunger. Unless she gets something to eat, almost faints. Sulph. has apparently cured epilepsy for me several times. Caused watery vomiting in some of the cases. Incredible as it may seem to those who have no experience with proving potentized remedies, I have frequently caused the most alarming symptoms with Sulphur high—a condition resembling paraplegia of the lower extremities. (Cured a case of vomiting, due to cerebral congestion from exposure to sun.) Highly curative in young persons with scrofulous ophthalmia. Cured two bad cases of amblyopia, dimness of sight; one in a girl, the other in a man. When he or she picked up a paper to read, the letters had a *double* instead of a single stroke or line. This is the language of the prover. Mist before the eyes, both had the symptom as if they were blinded for an instant with a dark cloud before the eyes. Caused and cured constipation as well as disposition to piles in nearly all the provers. Symptoms of the lower extremities given above were the most prominent.

### SYMPHYTUM-OFF.$^{5C}$.

Pains across his epigastrium from one side to the other; worse opposite the spleen and in walking; when sitting pain is severe about the navel; griping pain; headache sometimes in the occiput, and again in top of head, occasionally in forehead; indefinable headache all over the head. Menses stopped; great deal of headache. Feeling of weight in the forehead constantly. Considerable fever, which comes and goes often during the day. Often complains of coldness, cramp, and diarrhœa; nasal cavity sore, picking at the nose; rubs her eyes; inflamed ears, feels as if something was in them, stopped up, can't hear well, slight deafness; feeling miserably; generally weak and no desire or ability to be employed.

### TABACUM$^{67M}$.

Cured in the prover an unhealthy, pimply, sycotic skin, existing a long time; numbness in left upper extremity, side of head, and ear; all puffed up about the abdomen; left sterno-mastoid muscle very painful to touch, with drawing pains; at times crampy muscular pains in different parts of the body. In swallowing and after the food passes down an aching would occur between the clavicles, up the left side of the neck into the left ear; *feels as if the œsophagus was too small for food; always coughed after eating;* slight sore throat became well in one day after taking the medicine; hot hands, heat on top of head, urine high-colored; tongue coated; feels weak and fainty, only lasting a few minutes; nervousness; sleeplessness; sighing; anxiety in left chest mostly.

Cured in the prover a grunt-like clearing of the throat, coming on every few minutes; had been troubled off and on for years with it; caused anxiety and great restlessness, with fluttering sensation of the heart, and attending nausea. Verified this a great many times.

### TARTAR-EMET.$^{45M}$.

Caused inflamed lids, catarrhal conjunctivitis, soreness all over the chest. Promptly cured on the prover long-lasting dyspeptic symptoms, with loss of appetite, soreness on left hypochondrium. All the provers developed cough, which was constant and distressing; disposed to be loose; much expectoration.

### TELLURIUM$^{5C}$.

Neuralgia of the side of face and head, affecting the fifth pair of nerves; verified this symptom by cures; pain intermitting and severe; general neuralgic pains flying about and in various parts of the body, along course of the nerves.

### UVA-URSI.$^{10M}$.

Feels weak, nauseated, sore all over as if bruised; pain in chest, cold in head, throat tickles, feels like coughing continuously; nasal mucous membrane appears raw; symptoms of a severe cold; excess of mucus in throat; no appetite; cutting sensation in urethra on urination; often cured hoarseness.

### TEREBINTHINA$^{1M}$.

Highly curative in piles; causes and cures conjunctivitis; one of the most effective remedies in early stages of gonorrhœa, and as a remedy in gleet.

Produced painful, burning urination, disposition to urinate every ten minutes, third day of proving. Caused burning in the uterus with severe bearing-down pain; heat all over her; craves drink; complains of heat in pelvis; abdomen is fuller than usual. I have found this to be a most valuable remedy in checking the profuse bleeding occurring often in cases of very large uterine fibroids; nostrils sore; disposed to bleed; cured frequently bloody stools; disposition to bleed from mucous surfaces.

*Piles, bleeding and painful;* symptoms of cold in the head; hearing affected; own voice (and others) sounds unnatural; right ear has moist discharge; sounding and humming in left ear as of a seashell held to ear; can't tell who the person is who speaks unless sees him; talking loud is very painful to ears; irritation passed from right to left ear; ear symptoms only developed in a few provers.

After proving the remedy four days, caused smothering feeling; never comes on in day, only at night, or about eleven P. M.; on going to bed, smothering feeling referred to ensiform cartilage, but mostly to the larynx; obstruction apparently there, which comes on whenever he lies down; then has a tight cough for an hour or so; difficulty of expelling the urine; frequent urination; small quantity passed.

Have frequently cured with this remedy wetting the bed at night, involuntary stools, disposition to pick the nose, and worms; children's complaints; bronchitis in the very young.

### THERIDION-CURASS.$^{5C}$.

Frequent urination at night. Constipation, sore throat, lasting a week; chilly; bones feel sore; difficulty of swallowing from enlarged tonsils; water scanty and high-colored.

### TRIFOLIUM-ALBUM.$^{1M}$.

Giddy; head and face feel hot; severe dyspepsia; coated tongue; constipation; skin rough and dry.

### THUJA-OCCIDENT.$^{60M}$.

Loose bowels, four movements daily. Cured, in two or three weeks, warts on hands, existing fourteen years; hands covered with warts for years, cured in a few weeks; frequently cured long-lasting gleet, after gonorrhœa; troublesome, slight, long-lasting cough.

### TRIFOLIUM-PRAET.$^{10M}$.

*Complete aphonia*, choking sensation always comes on toward night; feels as if something was obstructing the throat and should come away. No sore throat; couldn't speak above a whisper; has a desire to clear her throat, but not sore; gums spongy and sore; hoarseness.

Have made repeated trials of this remedy in potentized and crude form in several varieties of cancer and without any good effect.

Relieved *constipation effectually;* bowels were loose for a long while after taking the medicine.

*Eyes weak* and watery.

Dull headache over both eyes; shooting neuralgic pain in the teeth; cramp in the left sterno-mastoid muscle; drew his head to one side for a good while, and only relieved by great heat and rubbing; working, gnawing sensation, with much gas in his stomach; saliva seems sour; bowels which were greatly constipated now regular; cleaned off a continually-coated tongue. Have often cured a common set of symptoms, bad breath, continually coated tongue, loss of appetite; food does not have proper taste; constipation; stools dark and hard; soreness in small intestines, with much flatulence.

### TRIGONOCEPHALUS-PISCIVORUS.

Poison of Trigonocephalus Piscivorus, 30th decimal, prepared by myself from specimens I caught on coast of Gulf of

Mexico. Produced profuse and long-lasting, watery, comparatively painless diarrhœa, and at times instant giddiness. Provers would fall in a faint or swoon and remain in that condition for a few moments. Some others would feel slightly nauseated and giddy. Great pallor. Highly curative in giddiness.

### TRILLIUM-PENDULUM$^{10M}$.

Shooting pains through the chest; symptoms of cold in the chest; aching of the muscles in general, as if from exposure to cold and dampness.

Violent cramp-like pain at the end of the sternum, very severe and continuous for some days; later, as it became better, a sensation of pressure and squeezing remain, with some difficulty of breathing because of it; cramp-like pains on various parts of the body; worse at night. Variable symptoms in other provers counted out. These appeared to be constant.

### TRITICUM$^{5C}$.

Soreness in the middle of the sternum; pain catches him every time he coughs; constant coughing; always blowing his nose; pain across the front of chest, low down; sore to touch and pressure. Nostrils clogged with mucus.

Soreness all along the sternum and in the stomach; pain runs through to the back from the sternum; itchy around the lower eyelids—they burn and sting; coughs a little, without expectoration. General soreness in chest.

Symptoms of cold in head with some cough; much sneezing, which strains him across the upper part of his chest; slightly hoarse, brings up with coughing some tough phlegm.

Soreness across the upper part of the chest in all the provers.

### TROMBIDIUM$^{15C}$.

Dryness in the nose; snuffles without discharge. Rheumatic-like pains in toes of left foot; sensation of general heat all over the body, with oppressed breathing. Joints of extremities feel sore generally.

### TUSSILAGO-FARFARA$^{1M}$.

Intolerable stupidity and sleepiness; impossible to keep awake; distended feeling from epigastrium downward; bowels much constipated.

### VALERIANA-OFFICINALIS$^{CM}$.

More than doubled the amount of urine usually passed; nothing peculiar about it other than being very light in color. This was the first prominent symptom. Great restlessness.

### VARIOLINUM$^{45M}$.

Has proven wonderfully successful in small-pox. I gave it to twenty cases at least during the epidemic of 1873, in Philadelphia, and where exhibited early it had, I believe, a markedly modifying and curative effect on the disease.

### VERBASCUM-THAPSUS$^{45M}$.

Quickly cured severe soreness in the pharynx, felt on swallowing. This has occurred often; guided by a similar symptom produced by it. Sensation of heat in epigastrium as if from dyspepsia.

Violent diarrhœa on the third day after taking the medicine; eighteen to twenty movements daily; griping; a great deal of pain as if pierced with a lance; pain in both cheek bones and above her eyesbrows; menses come on earlier; coughed a good deal; neuralgic pain in left ankle; cramps around the navel; seems as if the pain was caused by bowels becoming twisted; burning on passing water, which was voided often; heels hurt her severely; *great stiffness in left ankle joint;* more or less soreness and stiffness in the joints of the lower extremeties.

### WISTERIA$^{5C}$.

Very sharp pain across the small of the back, both sides; worse on walking; never had it before; would come on instantly and lame him.

### WOORARI$^{90M}$.

Nausea continually; vomits bile occasionally; feels very weak not disposed to walk about; very bitter, disagreeable taste; lips and body dusky at first, with fever, afterward prover becomes very red in the face; sensation of hammering in the head; *instant giddiness.* No perspiration visible, but general continuous fever, but not very great.

### ZINC-SULPH.$^{5C}$.

Had to arise in the night two or three times to pass water; mouth felt and was sore; tongue sore; general dull and listless feeling, with no appetite. Complained of having severe dyspepsia, like a load in epigastrium. No eructations.

### ZINC-OXIDE$^{2C}$.

Persistent tight cough mostly at night; dull pain in chest; free expectoration of tough mucus; nausea; mouth and tongue very sore; lips chapped in spots as large as the end of the finger; highly curative in certain forms of ulceration or inflammation of the cornea, particularly in scrofulous children with cough and dyspepsia. With disposition to ulceration of skin. Skin rough. Sore patches.

### ZINGIBER$^{CM}$.

Loose bowels and griping stools, movements which were previously dark and hard, are now light in color. Nausea. Constant eructations of wind—not particles of food.

